# Gamma-Irradiated Influenza A Virus Provides Adjuvant Activity to a Co-Administered Poorly Immunogenic SFV Vaccine in Mice

**DOI:** 10.3389/fimmu.2014.00267

**Published:** 2014-06-10

**Authors:** Rachelle Babb, Jennifer Chan, Jasmine E. Khairat, Yoichi Furuya, Mohammed Alsharifi

**Affiliations:** ^1^Vaccine Research Laboratory, School of Molecular and Biomedical Science, Centre for Molecular Pathology, The University of Adelaide, Adelaide, SA, Australia; ^2^Department of Immunology, The John Curtin School of Medical Research, Australian National University, Canberra, ACT, Australia

**Keywords:** influenza vaccines, combined vaccine, gamma-irradiation, adjuvants, antibodies

## Abstract

Many currently available inactivated vaccines require “adjuvants” to maximize the protective immune responses generated against the antigens of interest. Recent studies in mice with gamma-irradiated influenza A virus (γ-FLU) have shown its superior efficacy compared to other forms of inactivated FLU vaccines and its ability to induce both potent interferon type-I (IFN-I) responses and the IFN-I-associated partial lymphocyte activation. Commonly, IFN-I responses induced by adjuvants, combined in vaccine preparations, have been shown to effectively enhance the immunogenicity of the antigens of interest. Therefore, we investigated the potential adjuvant activity of γ-FLU and the possible effect on antibody responses against co-administrated antigens, using gamma-irradiated Semliki Forest virus (γ-SFV) as the experimental vaccine in mice. Our data show that co-vaccination with γ-FLU and γ-SFV resulted in enhanced SFV-specific antibody responses in terms of increased titers by sixfold and greater neutralization efficacy, when compared to vaccination with γ-SFV alone. This study provides promising evidence related to the possible use of γ-FLU as an adjuvant to poorly immunogenic vaccines without compromising the vaccine efficacy of γ-FLU.

## Introduction

Vaccines represent a fundamental aspect of current control strategies against infectious diseases. However, despite the remarkable success of many vaccines, there still remains many challenges in the field of vaccinology, such as generating effective vaccination strategies against notoriously difficult pathogens like hepatitis C virus and human immunodeficiency virus (1). Among the various types of currently used vaccines, live attenuated vaccines can mimic natural infections and consequently have been shown to be very effective in generating long-lived immunity. However, due to the high risk of conversion to their highly pathogenic form and the inability to develop live attenuated vaccines for many pathogens, inactivated vaccines such as inactivated whole viruses and purified antigens have typically been used as the main strategy for vaccine design. However, poor immunogenicity of many inactivated vaccines has severely affected their effectiveness. Therefore, inactivated vaccines often require “adjuvants” to enhance their immunogenicity. Importantly, most immunostimulatory adjuvants have been designed to potently stimulate innate signaling pathways through pattern recognition receptors (PRR) such as toll-like receptors (TLR) and cytosolic receptors (2–5). This ultimately leads to the induction of genes encoding for various immune-modulatory molecules including interferon type-I (IFN-I), which stimulate co-stimulatory molecule expression and antigen presentation (6).

Currently in vaccine design, greater attention is being drawn toward designing adjuvants to effectively boost the immune response toward existing vaccine preparations, which fail to induce sufficient immunity. The influence of IFN-I exogenously or through PRR stimulation has been shown to be very effective (7–9). We have previously reported the superiority of gamma-irradiated influenza virus (γ-FLU), compared to other flu vaccine formulations, in terms of inducing cross-protective immunity (10–12). We have also shown that γ-FLU similarly to its live form is capable of inducing potent IFN-I responses and the associated partial lymphocyte activation 24 h post-challenge (13, 14). Importantly, in contrast to gamma-irradiation of influenza, we have shown that gamma-irradiation of Semliki Forest virus (SFV) abrogates its ability to induce IFN-I responses (15). Therefore, we investigated the potential adjuvant activity of γ-FLU on co-administered γ-SFV as an experimental model for poor immunogenic vaccines. Here, we report that co-vaccination with γ-FLU and γ-SFV resulted in enhanced SFV-specific antibody titers “by six-folds” when compared to vaccination with γ-SFV alone. This enhancement in antibody titer was also associated with greater SFV neutralization efficacy and importantly; the vaccine efficacy of γ-FLU was not affected.

## Materials and Methods

### Ethics statement

This study was carried out in strict accordance with the recommendations in the Guide for the Care and Use of Laboratory Animals of The University of Adelaide. The protocol was approved by the Animal Ethics Committee at The University of Adelaide (Permit Number: S-2011/119).

### Viruses and cells

Avirulent SFV (A7 strain) was grown *in vitro* by infecting Vero cells using multiplicity of infection (MOI) of 0.1, and infected flasks were incubated for 24 h at 37°C in a humidified atmosphere with 5% CO_2_. Culture supernatants were then collected and clarified to remove cellular debris by centrifugation at 1500 rpm for 5 min. Virus titer was determined by plaque assay on Vero cells to be 3 × 10^8^ PFU/ml. For the vaccine preparation, SFV stock was concentrated using Millipore filtering devices with 100 kDa cut-off (Millipore) and centrifugation at 2000 rpm for 1 h at 4°C using Eppendorf bench top centrifuge. Virus titer of the concentrated SFV was determined by plaque assay on Vero cells to be 5 × 10^8^ PFU/ml.

The influenza type A virus, A/PR/8 [(A/Puerto Rico/8/34 (H1N1)], was grown in 10-day-old embryonated chicken eggs (HiChick, SA, Australia). Each egg was injected with 0.1 ml normal saline containing 1 hemagglutination unit (HAU) of virus, incubated for 48 h at 37°C, and then held at 4°C overnight. The amniotic/allantoic fluids were then harvested, pooled, clarified, and stored at −80°C. Gamma-irradiated A/PR8 vaccine preparations were previously prepared by Dr. Furuya at ANU. Briefly, concentrated virus stocks were prepared using chick erythrocytes as previously described (16). Infectious allantoic fluid was incubated with chicken red blood cells (cRBCs) for 45 min at 4°C allowing the hemagglutinin to bind to erythrocytes, and then centrifuged (4°C, 1500 rpm, 10 min) to remove the allantoic fluid supernatant. The pellets were resuspended in normal saline, incubated for 1 h at 37°C to release the RBCs from the virus, and then centrifuged to remove the erythrocytes and the supernatant containing the virus collected. The titer of the concentrated A/PR8 virus stock (9 × 10^8^ TCID_50_/ml) was determined by TCID50 assay (17).

### Virus inactivation

Concentrated virus stocks were inactivated by exposure to gamma-irradiation from a ^60^Co source [Australian Nuclear Science and Technology Organization (ANSTO) at Lucas Heights/NSW]. A/PR8 and SFV received a dose of 10 and 50 kGy, respectively, and they were kept frozen on dry ice during gamma-irradiation. Sterility was tested by two independent methods: plaque assay using MDCK (for A/PR8) or Vero cells (for SFV); and by inoculating embryonated eggs (for A/PR8). The detection limit of our plaque assay is 10 PFU/ml and no plaque forming unit was detected for the irradiated samples. These tests confirmed sterility of inactivated stocks. In addition, we have estimated the minimum inoculum required to cause a positive infection in embryonated eggs and found that the minimum egg infectious dose that causes detectable HA titers in the allantoic fluid after 2 days of incubation is 0.1 TCID50/egg. Embryonated eggs were inoculated with 100 ml of inactivated preparations per egg and incubated for 2 days at 37°C and the allantoic fluid of individual eggs was harvested and tested for virus replication using HA assays. HA titers were negative in the allantoic fluid of these eggs, which illustrates a complete loss of virus infectivity in our inactivated preparations.

### Mice and treatments

Wild-type C57B/6 mice (9–10-week-old) were bred under specific pathogen-free conditions and supplied by the Animal Laboratory Services at the University of Adelaide, SA, Australia.

In general, vaccine preparations were diluted using 10-fold serial dilutions and each mouse was injected in the tail vein with 200 μl of the relevant virus or vaccine preparation. The following doses were used: live SFV (10^7^ PFU/mouse), γ-SFV (either 10^6^, 10^7^, or 10^8^ PFU equivalent/mouse), and γ-FLU (10^4^, 10^5^ TCID50 equivalent/mouse). Refer to text for specific doses used in each experiment. For co-vaccination, the two vaccine preparations were mixed thoroughly in the same tube and administered as a single injection into experimental animals. Vaccination doses are expressed PFU or TCID50 equivalent. In addition, in some experiments Poly(I:C) was injected intravenously at a dose of 150 μg in 200 μl of PBS per animal as previously reported (18).

### Antibody analysis

Semliki Forest virus-specific and FLU-specific antibody responses in serum samples were determined by enzyme-linked immunosorbent assay (ELISA). In brief, Maxisorp plates were coated with concentrated SFV or FLU viral antigen diluted in bicarbonate coating buffer (Na_2_CO_3_, NaHCO_3_, water at pH 9.6) and incubated overnight at room temperature. Non-specific protein binding sites were then blocked with PBS containing 2% skim milk powder for 2 h at room temperature. Fifty microliter volumes of serially diluted serum samples were added to the appropriate wells for 2 h at room temperature followed by the addition of horse radish peroxidase conjugated goat anti-mouse IgG (Thermo Scientific) at room temperature for 2 h. Plates were developed using TMB peroxidase substrate in the dark for 30 min and the reaction was stopped with 2 mol H_2_SO_4_. Absorbance was measured at 450 nm using a Microplate ELISA reader (Bio-Tek Instruments).

### Neutralization assays

Plaque reduction assay modified from (19) was used to analyze SFV neutralization. Twenty-four well tissue culture plates were seeded with 1.5 × 10^5^ Vero cells/well and incubated overnight at 37°C in a humidified atmosphere with 5% CO_2_. Aliquots of serum samples from control and vaccinated animals were incubated at 56°C for 30 min to inactivate complements and serially diluted using EMEM media without FCS. Diluted samples were mixed with equivalent amount of DMEM media containing 100 PFU of SFV. Mixtures (sera and virus) were incubated for 1.5 h at 37°C and then used to infect confluent Vero cell monolayer’s (in triplicate) prepared earlier. Initially, culturing media was removed from each well prior to addition of virus/serum mixture. Plates were then incubated for 2 h at 37°C to allow infection of monolayers. Following incubation, the infecting mixture was removed and an agar overlay containing 50% of 1.8% Bacto-Agar, 40% DMEM media, 10% FCS, and 0.002% Fungizone was added to each well and plates were incubated for 3 days at 37°C, 5% CO_2_. Following incubation, cells were fixed with 5% formalin for 1 h at room temperature. The overlay was then carefully removed and cell monolayers were stained with 0.2% crystal violet. Plaques were enumerated to determine the effect of the serum on virus infectivity.

In addition, a hemagglutination inhibition assay (HAI) was used to test the influenza-specific antibody responses, as previously described (17). Aliquots of sera were incubated at 56°C to inactivate complements for 30 min, then diluted in PBS containing 1% RBCs, and left for another 30 min incubation at room temperature. RBCs within samples were then pelleted by centrifugation at 1400 rpm using a microcentrifuge and supernatants were collected. Twofold serial dilutions were performed using a 96-round well-bottom plate. Fifty microliter of diluted virus (FLU) at a concentration of 80 HAU/ml was then added to each dilution of sera and incubated for 30 min at room temperature. PBS containing 1% RBCs was then added to each well and incubated at 4°C. Results were analyzed 24 h later and neutralizing antibodies in each dilution was determined by the presence of a pellet of RBCs at the bottom of the wells.

### Statistical analysis

Results were expressed as mean ± SEM. Statistical significance among samples was calculated using an unpaired Student’s *t*-test. *P* values <0.05 were considered statistically significant.

## Results

### Antibody responses induced by γ-SFV

**Figure 1:**
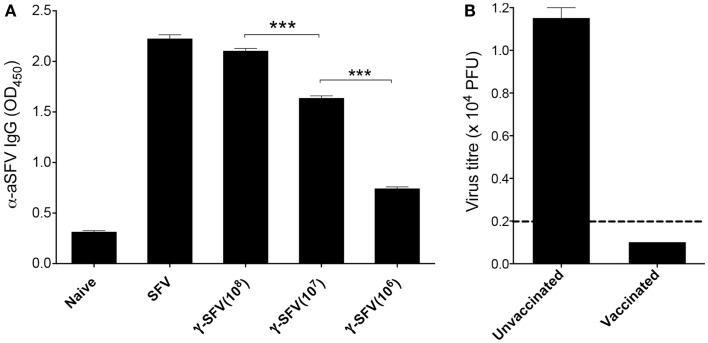
**γ-SFV vaccine induces protective antibody responses**. **(A)** Mice were injected i.v. with SFV (10^7^ PFU) or variable doses of γ-SFV (10^6^, 10^7^, or 10^8^ PFU equivalent/mouse). Twenty days post-injection, serum SFV-specific IgG levels were measured by direct ELISA using a serum dilution of 1/200. Serum from naive mice served as the negative control. **(B)** Mice were vaccinated i.v. with γ-SFV (10^7^ PFU equivalent/mouse) and challenged 14 days post-vaccination with SFV (10^7^ PFU). Twenty four hours post-challenge, serum SFV titers were determined by plaque assay. Un-vaccinated naive mice infected with SFV served as control. Results are presented as mean ± SEM (*n* = 3) and dashed line represents our assay detection limit of 100 PFU, ***p < 0.001.

Avirulent SFV causes asymptomatic infection characterized by high-titer viremia in adult mice (20, 21), and effective viral clearance has been attributed to rapid antibody responses generated by the host (22). Interestingly, primary cytotoxic T cell responses have been shown to be associated with MHC-I haplotype and restricted to H-2^k^ haplotype expressing mice (23). Particularly, C57/B6 mice (H-2^b^ haplotype) have been classified as cytotoxic T cell non-responders and therefore were used in the study. To confirm that the γ-SFV vaccination strategies used in this study can generate effective antibody responses, serum SFV-specific IgG levels were measured 20 days post-infection with live SFV (10^7^ PFU/mouse) or vaccination with variable doses of γ-SFV (10^6^, 10^7^, or 10^8^ PFU equivalent/mouse). Our data illustrate that vaccination with γ-SFV induces high levels of SFV-specific IgG in the serum of vaccinated mice in a dose-dependent manner (Figure 1A).

To determine if the detected antibody responses are protective, mice were vaccinated with γ-SFV (10^7^ equivalent PFU/mouse) and challenged 21 days later with live SFV (10^7^ PFU/mouse). Twenty-four hours post-challenge, serum samples were tested for virus infectivity by plaque assay. No viral infectivity was detected in all serum samples from previously vaccinated mice in contrast to the high viremia observed in un-vaccinated control mice following a challenge with live SFV (Figure 1B).

**Figure 2:**
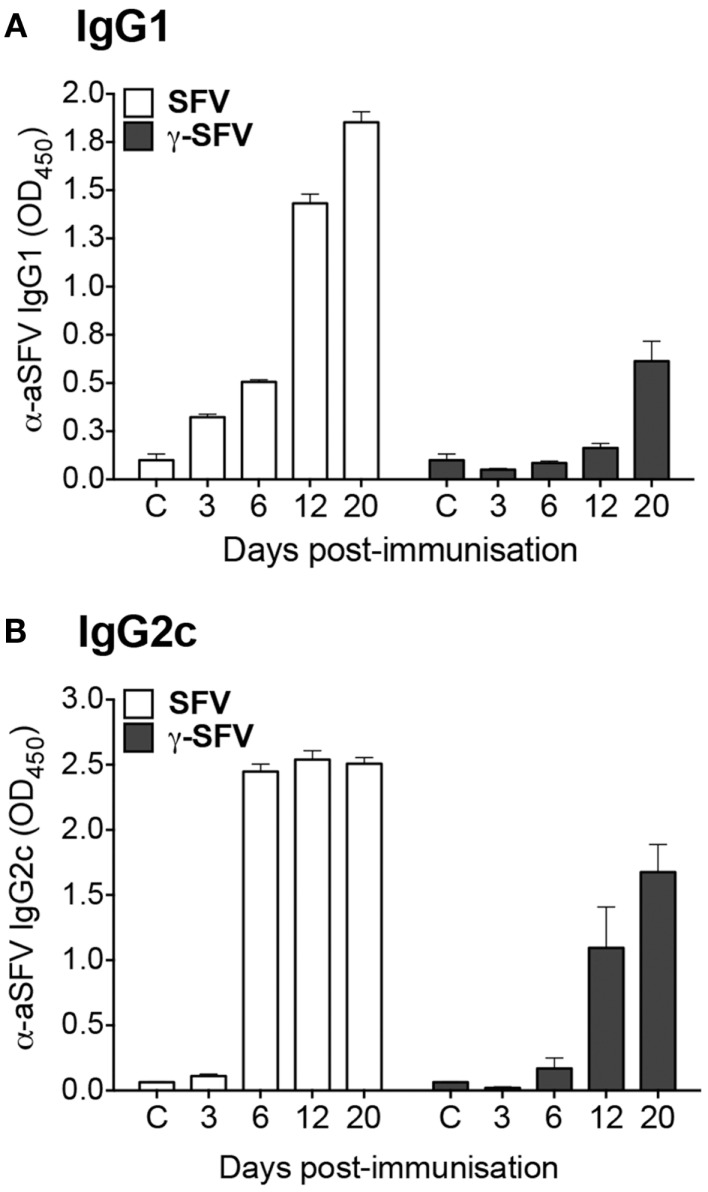
**Immunization with γ-SFV promotes lower antibody levels compared to SFV**. Mice were injected i.v. with SFV (10^7^ PFU) or γ-SFV (10^7^ PFU equivalent/mouse). Serum SFV-specific IgG1 **(A)** and IgG2c **(B)** levels were measured at 3, 6, 12, and 20 days post-immunization by direct ELISA at a serum dilution of 1/200. Sera from naive mice served as negative controls (c). Results are presented as mean ± SEM (*n* = 3), **p < 0.01.

It has been previously shown that live SFV infection induces IFN-I and as a consequence promotes Th1 antibody isotype switching (20). Considering the inability of γ-SFV to induce detectable levels of IFN-I (13, 15), the level of IgG isotypes induced by live SFV vs. γ-SFV was investigated. Mice were infected with SFV (10^7^ PFU/mouse) or vaccinated with γ-SFV (10^7^ equivalent PFU/mouse) and serum SFV-specific IgG1 and IgG2c levels were measured over a time course. Both IgG1 and IgG2c levels appeared to be lower following vaccination with γ-SFV in comparison to SFV (Figures 2A,B).

### The effect of co-administration of γ-FLU and γ-SFV on SFV-specific antibody responses

**Figure 3:**
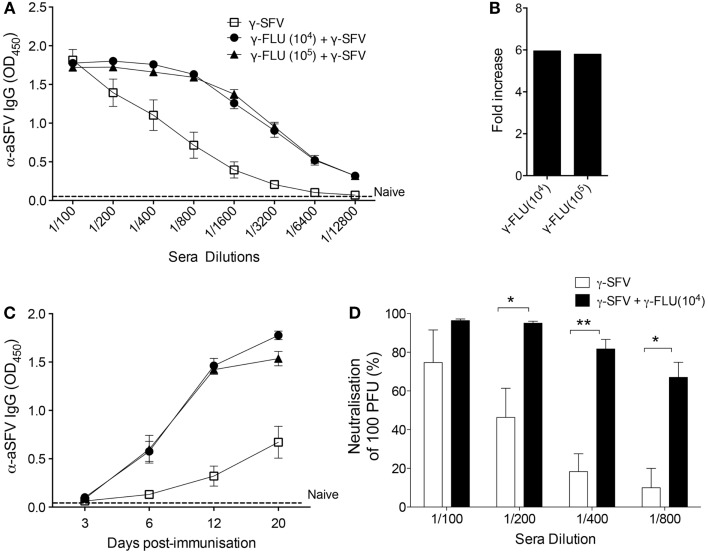
**Co-administration of γ-FLU and γ-SFV enhances SFV-specific antibody responses**. Mice were injected i.v. with a single dose of γ-SFV (10^7^ PFU equivalent/mouse) or co-injected with various doses of γ-FLU (10^4^, 10^5^ TCID50 equivalent/mouse). **(A)** Serum SFV-specific IgG concentrations 20 days post-vaccination were analyzed at twofold sera dilutions by direct ELISA and serum from naive mice served as the negative control (naïve). **(B)** Folds increase in SFV-specific IgG antibody titers based on the absorbent value of 0.5 for the tested serum dilutions **(A)**. **(C)** Serum SFV-specific IgG levels were analyzed by direct ELISA using a serum dilution of 1/800 at 3, 6, 12, and 20 days post-vaccination. **(D)** Neutralization of SFV by the immune sera collected at day 20 post-vaccination as determined by plaque reduction assay. Data represent mean ± SEM (*n* = 3), *p < 0.05, **p < 0.01.

Considering the potent IFN-I responses induced by γ-FLU (13, 14), we investigated the effect of γ-FLU and γ-SFV co-administration on SFV-specific antibody responses. Mice were co-injected with γ-SFV (10^7^ PFU equivalent/mouse) and γ-FLU (10^4^ or 10^5^ equivalent TCID50/mouse) and serum SFV-specific IgG levels were measured at day 20 post-vaccination. Our data illustrate the significant enhancement (~six-folds) of SFV-specific IgG levels in mice co-injected with both γ-SFV and γ-FLU compared to mice vaccinated with γ-SFV alone (*p* < 0.05) (Figures 3A,B). In addition, we investigated the kinetics of SFV-specific antibody responses post co-administration of γ-SFV and γ-FLU. Similarly, our data illustrate the consistent enhancement of SFV-specific antibody responses at all tested time points (*p* < 0.05) (Figure 3C).

To determine whether enhancement of SFV-specific antibody responses mediated by co-administration of γ-FLU coincides with enhanced SFV neutralization, serial dilutions of the immune sera were tested for their ability to neutralize 100 PFU of live SFV *in vitro* using a Vero cells based plaque-inhibition assay. Our results clearly illustrate that while immune sera from γ-SFV alone vaccinated mice have high neutralization activity at 1/100 dilution, this neutralization activity decreased remarkably with every serial dilution to reach ~10% activity at 1/800 serial dilution. In contrast, immune sera from γ-SFV and γ-FLU co-vaccinated mice show significantly higher neutralization values for all tested sera dilutions when compared to sera from γ-SFV alone vaccinated mice (Figure 3D).

**Figure 4:**
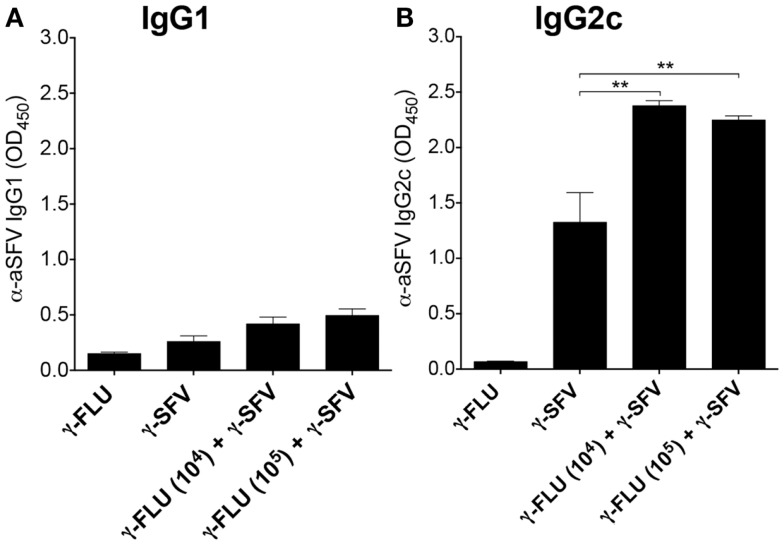
**Co-administration of γ-FLU and γ-SFV enhances SFV-specific IgG2c responses**. Mice were injected i.v. with a single dose of γ-SFV (10^7^ PFU equivalent/mouse) or co-injected with various doses of γ-FLU (10^4^, 10^5^ TCID50 equivalent/mouse). SFV-specific IgG1 **(A)** and IgG2c **(B)** levels in the serum at day 20 post-vaccination were analyzed by direct ELISA using a serum dilution of 1/200. Sera from γ-FLU vaccinated mice served as negative controls. Data represent mean ± SEM (*n* = 3) **p < 0.01.

Next, we investigated the effect of γ-FLU and γ-SFV co-administration on SFV-specific IgG2c and IgG1 levels and whether co-vaccination may promote a particular IgG isotype. Our data illustrates that co-administration of γ-FLU and γ-SFV resulted in significantly enhanced IgG2c levels compared to vaccination with γ-SFV alone (*p* < 0.01). However, co-vaccination did not lead to significant enhancement in IgG1 responses as the detected SFV-specific IgG1 levels appeared similar in both vaccinated groups (Figures 4A,B).

### The effect of co-vaccination on FLU-specific antibody responses

**Figure 5:**
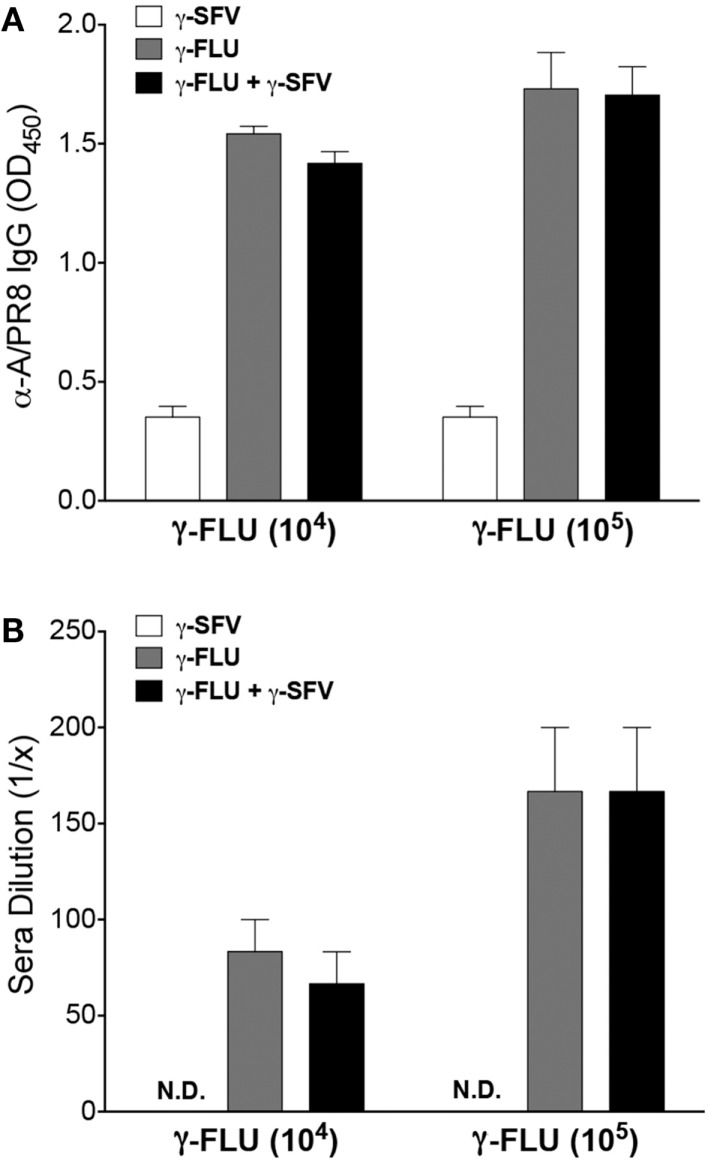
**The effect of co-vaccination on FLU-specific antibody responses**. **(A)** Mice were injected i.v. with γ-FLU (10^4^ or 10^5^ TCID50 equivalent/mouse) or co-injected with γ-SFV(10^7^ PFU equivalent/mouse) and serum FLU-specific IgG concentrations were analyzed at day 20 post-vaccination by direct ELISA using a serum dilution of 1/200. Sera from γ-SFV injected mice served as negative controls. **(B)** HA inhibition assay showing dilution of the immune sera capable of inhibiting the hemagglutinating activity of 80 HAU of A/PR8. Results are presented as mean ± SEM (*n* = 3).

Vaccination with γ-FLU has been shown to elicit homotypic neutralizing antibody responses (10, 11). To determine whether the co-administration of γ-FLU and γ-SFV affects the host’s ability to generate effective FLU-specific humoral responses, we investigated FLU-specific IgG responses at day 20 post-vaccination. Our data indicates that co-vaccination did not suppress the induction of FLU-specific IgG responses induced by γ-FLU (Figure 5A). We have also tested the neutralizing efficacy of FLU-specific antibodies using HA inhibition assay and our data illustrated that the hemagglutinating activity of 80 HAU of A/PR8 was inhibited at similar levels by immune sera from mice vaccinated with γ-FLU alone or mice co-vaccinated with γ-FLU and γ-SFV (Figure 5B).

### The effect of an IFN-I inducing adjuvant on the immunogenicity of γ-SFV

**Figure 6:**
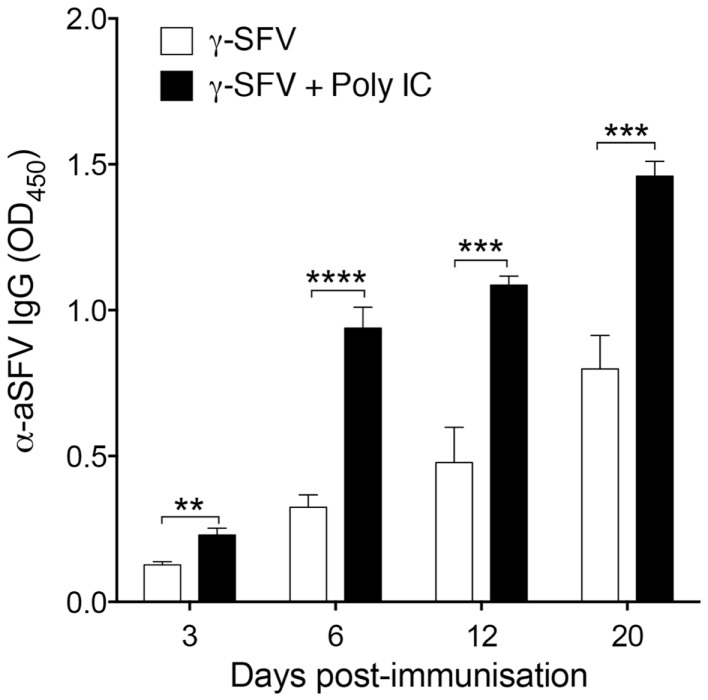
**Co-administration of Poly(I:C) and γ-SFV enhances SFV-specific IgG levels**. Mice were vaccinated i.v. with γ-SFV (10^7^ PFU equivalent/mouse) or co-injected with poly (I:C) (150 μg). Total SFV-specific IgG levels in the serum were analyzed at day 3, 6, 12, and 20 post-vaccination using direct ELISA using a serum dilution of 1/200. Sera from naive mice served as negative controls. Results are presented as mean ± SEM (*n* = 3) (*) denotes statistical significance, **p < 0.01, ***p < 0.001.

In line with the common approaches used for adjuvant design, the adjuvant activity of γ-FLU is expected to be related to its ability to induce potent IFN-I responses and the associated IFN-I-mediated partial lymphocyte activation (13). To illustrate the effect of an IFN-I inducing adjuvant on the immunogenicity of γ-SFV, we evaluated the effect of poly(I:C) and γ-SFV co-administration on SFV-specific antibody responses. The ability of poly(I:C) to induce IFN-I and its potential use as an adjuvant to enhance humoral responses toward poorly immunogenic proteins has been well-documented (4, 7, 24). Therefore, mice were vaccinated with γ-SFV with or without co-injection of poly(I:C) and total SFV-specific IgG levels in the immune sera were analyzed at days 3, 6, 12, and 20 post-vaccination. Our data illustrate that co-injection of poly(I:C) and γ-SFV resulted in a significant enhancement in the level of SFV-specific IgG titers in the serum at all time points compared to the injection of γ-SFV alone (Figure 6).

## Discussion

There has always been an increased demand for safe and effective vaccines to reduce the morbidity and mortality associated with particular viral infections. Non-living antigens are often employed in vaccine strategies and many are poor immunogens (2). We have reported previously the efficacy of γ-FLU to generate protective immunity upon homotypic and heterosubtypic influenza A virus challenges (10, 25). In addition, we have demonstrated the ability of the γ-FLU vaccine to induce potent IFN-I responses and the associated partial systemic lymphocyte activation (13, 14). It has been illustrated previously that IFN-I plays a very influential role in the development of B lymphocytes and consequently antibody production (26, 27). In addition, we have reported that γ-SFV, in contrast to live SFV, does not induce detectable levels of IFN-I (13, 15). Therefore, we used γ-SFV vaccine as an experimental model to test the adjuvant activity of γ-FLU.

In general, adjuvants are often used to achieve qualitative/quantitative differences in the immune responses that may include increasing the speed of an immunological response, which is important especially during pandemic outbreaks (28, 29). Adjuvants have also been employed to promote specific types of immunity, which may not be efficiently generated by the non-adjuvanted antigens, i.e., Th1 vs. Th2 cells, CD8^+^ vs. CD4^+^ cells, and specific types of antibody isotypes (2). Our data clearly show that co-administration of γ-SFV with γ-FLU amplified SFV-specific IgG levels with an overall enhancement of ~six-folds. The enhanced titers observed at day 6 post co-vaccination were equal to the titers detected at day 20 following vaccination with γ-SFV alone. Thus, confirming an earlier induction and amplification of SFV humoral responses. We have also shown this enhancement to be associated with increased efficiency in virus neutralization. Furthermore, our data illustrate that γ-FLU promotes enhancement of type-1 antibody response to co-administered γ-SFV, as illustrated by the significant increase in IgG2c but not IgG1. This outcome is commonly desired within vaccine development due to the competent functions of IgG2a during antigen clearance relative to IgG1 (30–32).

In line with the many approaches used for adjuvant design, IFN-I responses induced by γ-FLU may have played an important role in the observed adjuvant activity of γ-FLU. To evaluate the role of IFN-I, the adjuvant activity of Poly IC on antibody responses to γ-SFV was investigated. The enhancement of humoral responses against poorly immunogenic proteins using Poly I:C is well-documented (4, 7, 24). Poly IC, is a synthetic analog of dsRNA, which is commonly used to stimulate TLR3 and MDA-5 to induce IFN-I and other Th1 priming cytokines such as IL-12 (8, 33, 34). Consistent with previous reports, our data show that co-immunization with Poly IC and γ-SFV resulted in significantly enhanced SFV-specific IgG titers relative to titers observed following vaccination with γ-SFV alone. While this suggest a possible role of IFN-I, more work will be conducted to analyze the underline mechanisms for the adjuvant activity of γ-FLU.

The ultimate goal of using γ-FLU as an adjuvant is to exploit the efficacy of the γ-FLU-mediated immune response toward combined vaccines, in addition to conferring protection against the influenza virus. Our results show that co-administration of γ-FLU and γ-SFV did not affect the titers of FLU-specific IgG and did not affect the neutralizing activity of FLU-specific antibodies. Therefore, humoral responses generated against FLU antigens were not hindered when a second vaccine is present in the environment. Overall, this study is a proof-of-concept illustrating that γ-FLU, a whole virus killed influenza vaccine, can potentially be employed as an adjuvant to increase the quality and magnitude of immune responses toward co-administered less immunogenic vaccines. Future studies will investigate the clinical relevance of the γ-FLU-based combined vaccination strategy, particularly in relation to intranasal and intramuscular routes of administration. Future studies will also examine the effect of co-administered vaccines on the ability of γ-FLU to induce cross-protective immunity.

## Conflict of Interest Statement

The authors declare that the research was conducted in the absence of any commercial or financial relationships that could be construed as a potential conflict of interest.
